# Engineering Enzymes for Environmental Sustainability

**DOI:** 10.1002/anie.202309305

**Published:** 2023-10-05

**Authors:** Emily Radley, John Davidson, Jake Foster, Richard Obexer, Elizabeth L. Bell, Anthony P. Green

**Affiliations:** ^1^ Department of Chemistry & Manchester Institute of Biotechnology The University of Manchester 131 Princess Street Manchester M1 7DN UK; ^2^ Renewable Resources and Enabling Sciences Center National Renewable Energy Laboratory Golden CO USA; ^3^ BOTTLE Consortium Golden CO USA

**Keywords:** Biocatalysis, Directed Evolution, Enzyme Engineering, Sustainability

## Abstract

The development and implementation of sustainable catalytic technologies is key to delivering our net‐zero targets. Here we review how engineered enzymes, with a focus on those developed using directed evolution, can be deployed to improve the sustainability of numerous processes and help to conserve our environment. Efficient and robust biocatalysts have been engineered to capture carbon dioxide (CO_2_) and have been embedded into new efficient metabolic CO_2_ fixation pathways. Enzymes have been refined for bioremediation, enhancing their ability to degrade toxic and harmful pollutants. Biocatalytic recycling is gaining momentum, with engineered cutinases and PETases developed for the depolymerization of the abundant plastic, polyethylene terephthalate (PET). Finally, biocatalytic approaches for accessing petroleum‐based feedstocks and chemicals are expanding, using optimized enzymes to convert plant biomass into biofuels or other high value products. Through these examples, we hope to illustrate how enzyme engineering and biocatalysis can contribute to the development of cleaner and more efficient chemical industry.

## Graphical abstract

This review highlights how engineered enzymes have been developed and implemented to help address environmental challenges. Topics include the use of engineered enzymes for improving carbon capture and utilisation, bioremediation, plastic deconstruction, and renewable feedstock generation. Successes, challenges, and opportunities for future enzyme engineering campaigns to improve environmental sustainability are discussed.

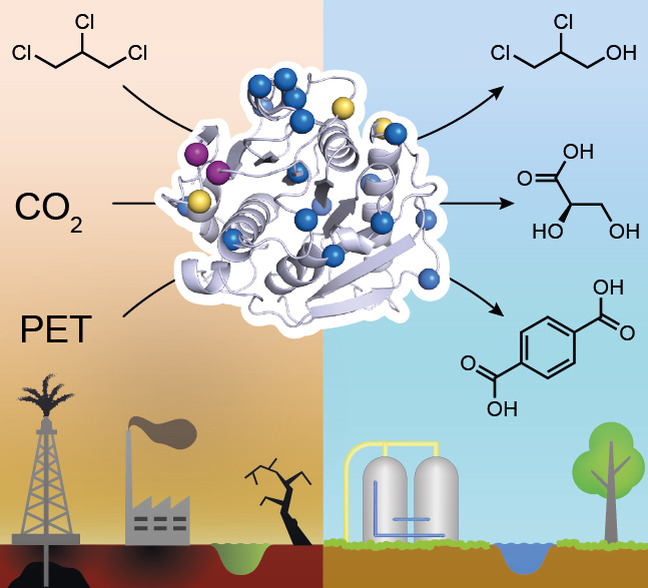

## 1 Introduction

The global population is currently over 8 billion and is projected to rise to 9.7 billion by 2050.^[1]^ Such growth raises major concerns over how we can promote environmental sustainability whilst continuing to fulfil the population's needs. Human activities have placed unprecedented demands on our planet and have led us towards an environmental crisis. Our over‐reliance on fossil fuels to produce the energy, chemicals, and materials essential for modern living has led to an unsustainable rise in greenhouse gas emissions. Increased demand for food is also driving carbon intensive agricultural processes to proliferate, contributing to a worrying rise in deforestation and massive biodiversity losses. Toxic chemicals that are harmful to health are all too often released and left in the environment,^[2]^ either because of unintentional leakages or as part of normal accepted operating processes. In addition, a global appetite for materials and packaging is leading to a damaging accumulation of environmentally recalcitrant waste plastics and further exacerbation of global warming, with the synthesis of single use plastics from fossil fuels accounting for 450 million metric tons of CO_2_ emissions in 2021 alone.^[3]^ As the population rises, unsustainable consumption patterns of fuels and products will intensify current problems.^[4]^ Issues are further complicated as solutions intended to mitigate environmental issues can sometimes lead to unintended negative consequences.^[5]^

**Figure 1:**
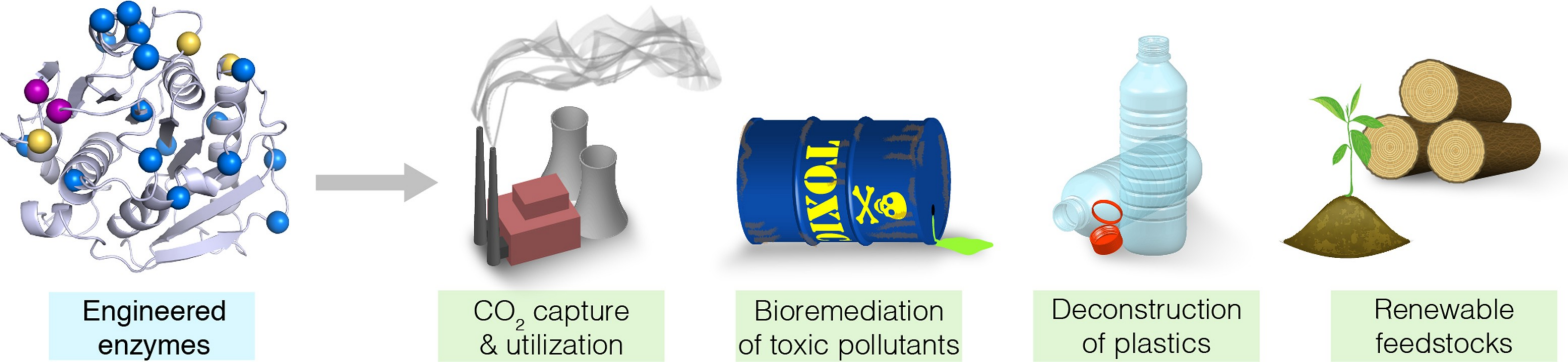
Schematic overview of the topics covered in this review.

Considering these challenges, there is clear need for fundamental changes across the chemical and energy sectors to reduce their environmental footprint and ensure the sustainability of our ecosystems for future generations. One technology that is already widely adopted by major chemical companies and will be a key component of a sustainable chemical industry is biocatalysis, whereby natural or engineered enzymes are used to effect chemical transformations.[^6^, ^7^] Enzymes are made from renewable feedstocks, are biodegradable, can operate under ambient reaction conditions, and promote reactions with remarkable efficiencies and selectivities. Furthermore, multiple enzymes can often be combined into “one‐pot” multi‐step cascades to allow complex chemical conversions to be achieved with dramatically improved step‐economy, leading to increased productivity and reductions in solvent usage and energy inputs.^[8]^ A key feature of enzymes that has contributed to their widespread implementation is their engineerability. Modern protein engineering methods mean that we are no longer limited to natural enzymes when designing biocatalytic processes. Instead, enzymes can now be specifically tailored to meet the needs of target applications.^[9]^ In this review, following a brief introduction to enzyme engineering with a focus on directed evolution, we will illustrate key examples of where enzymes have been developed to address environmental challenges (Figure 1). The article is not intended to provide comprehensive summary of all activities within the field, but rather to illustrate through selected examples the potential of enzyme engineering to positively impact environmental sustainability.

## 2 Enzyme engineering and directed evolution

**Figure 2:**
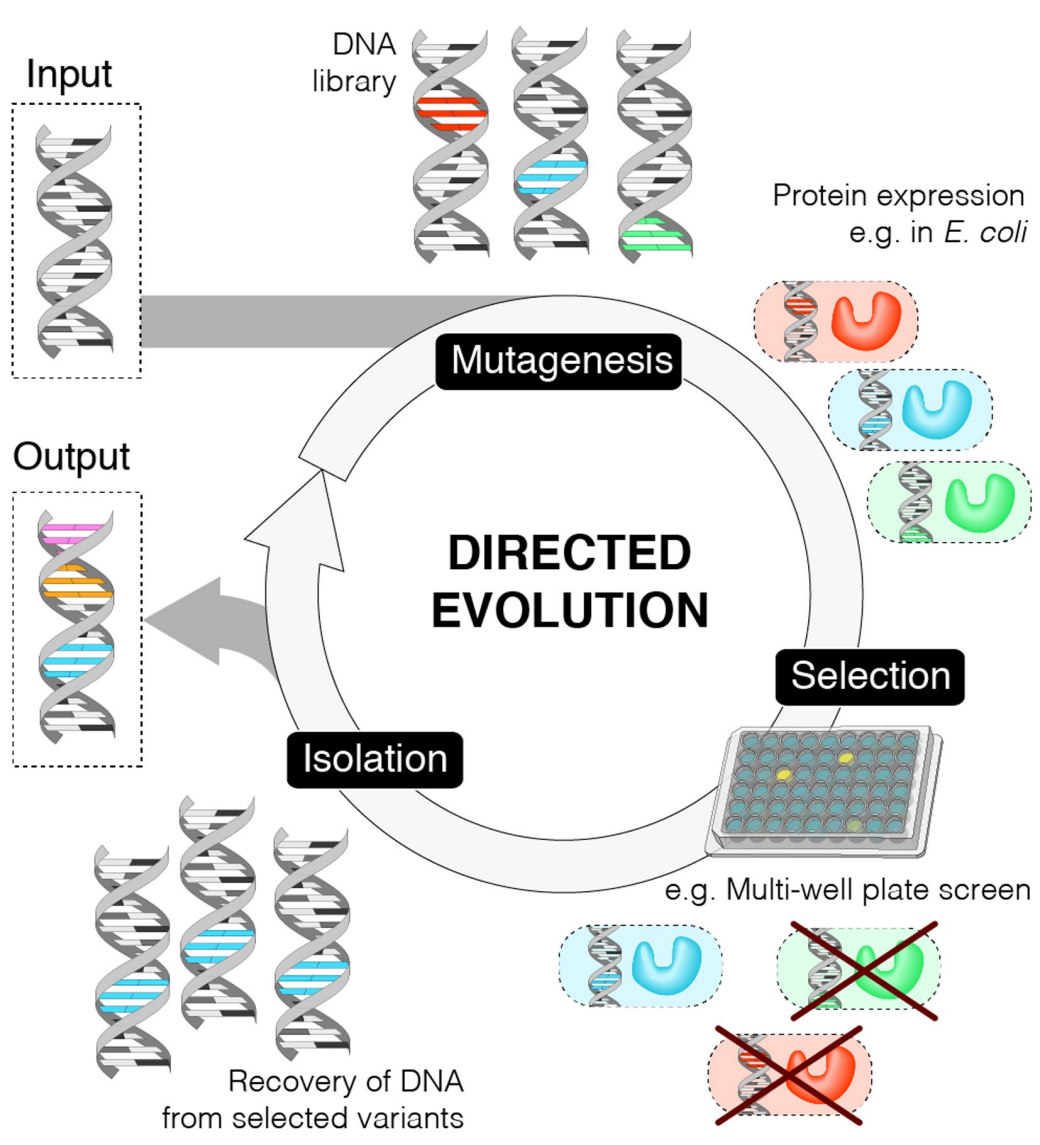
Directed Evolution. Directed evolution is comprised of three main steps: mutagenesis, selection of desired phenotypes, and isolation of the selected variants. Starting from the parent gene (input) the sequence is diversified e.g. by error prone PCR. Typically, the resulting DNA library is then transformed into bacteria for protein expression. Variants with desired properties can be identified (e.g. using a multi‐well plate assay) and isolated. The recovered genes serve as the input for the subsequent round of evolution. This cycle is iterated until the desired phenotypic activity is reached, typically resulting in accumulation of several mutations (output).

Wild‐type enzymes are often not suitable for a target application and must first be optimized to improve their properties.[^9^, ^10^] Enzymes can be engineered to increase substrate specificity or to broaden their substrate range, to improve selectivity and kinetic parameters, to increase their tolerance to immobilisation, and to enhance stability under process relevant conditions such as high substrate loadings, elevated temperatures or the presence of organic co‐solvents. Enzyme engineering can also be used to enhance promiscuous activities to unlock new catalytic functions.[^11^, ^12^] Directed evolution is a versatile and widely implemented strategy for enzyme engineering, mimicking Darwinian evolution under laboratory conditions and timescales (Figure 2).[^13^, ^14^] Using this approach, multiple enzyme properties can be optimized in parallel, even in the absence of detailed knowledge of the enzyme structure and mechanism. In some cases, directed evolution is used in conjunction with computational algorithms, such as protein sequence activity relationships (ProSAR) and those based on machine learning (ML), to more efficiently navigate sequence space and reduce the screening burden.[^15^, ^16^, ^17^, ^18^] Similarly, computational tools have also proven valuable for improving protein stability, rationally re‐engineering substrate binding pockets or even imparting new catalytic functions that can be subsequently refined through evolution.[^19^, ^20^, ^21^, ^22^, ^23^]

The directed evolution cycle involves iterative rounds of DNA library generation, gene expression and screening of enzyme library members. Following identification of a suitable starting template, DNA libraries are generated using standard molecular biology techniques, such as random or site saturation mutagenesis. Transforming cells with DNA libraries leads to spatial separation of library members and establishes a link between genotype and phenotype. This link must be maintained during protein production and screening to allow characterization of individual library members. Single colonies are commonly arrayed into multi‐well plates for protein production and screening, where they can be evaluated using a wide range of chromatographic, spectrophotometric, and spectroscopic techniques. In some cases, more specialized screening and selection‐based approaches can be used to increase throughput and allow more extensive exploration of protein sequence space. However, such methods typically require desired enzyme activity to be linked to a fluorescent output or to cell viability which is not possible in many cases.^[24]^ Following library evaluation, the top performing variants are isolated and characterized by DNA sequencing and serve as templates for subsequent rounds of evolution.

## 3 CO_2_ capture and utilisation

CO_2_ is released as a by‐product in many key industries, from energy production to agriculture, and is a major driver of global warming. To mitigate climate change, we need to reduce the amount of CO_2_ released into the atmosphere by industrial processes. One potential solution to curtail these emissions is to implement carbon capture and sequestration (CCS) technologies on coal and natural gas fired power plants, which are amongst the leading producers of anthropogenic CO_2_ emissions. One of the most developed CCS technologies makes use of an amine solvent to remove CO_2_ from the flue gas.^[25]^ However, while this approach offers fast CO_2_ absorption kinetics, a large amount of energy is needed to release the captured CO_2_ and regenerate the solvent.^[26]^ Switching to an aqueous amine solvent with a low heat of desorption can dramatically reduce the energy needed for solvent regeneration, but unfortunately such solvents suffer from prohibitively slow CO_2_ absorption kinetics.

**Figure 3:**
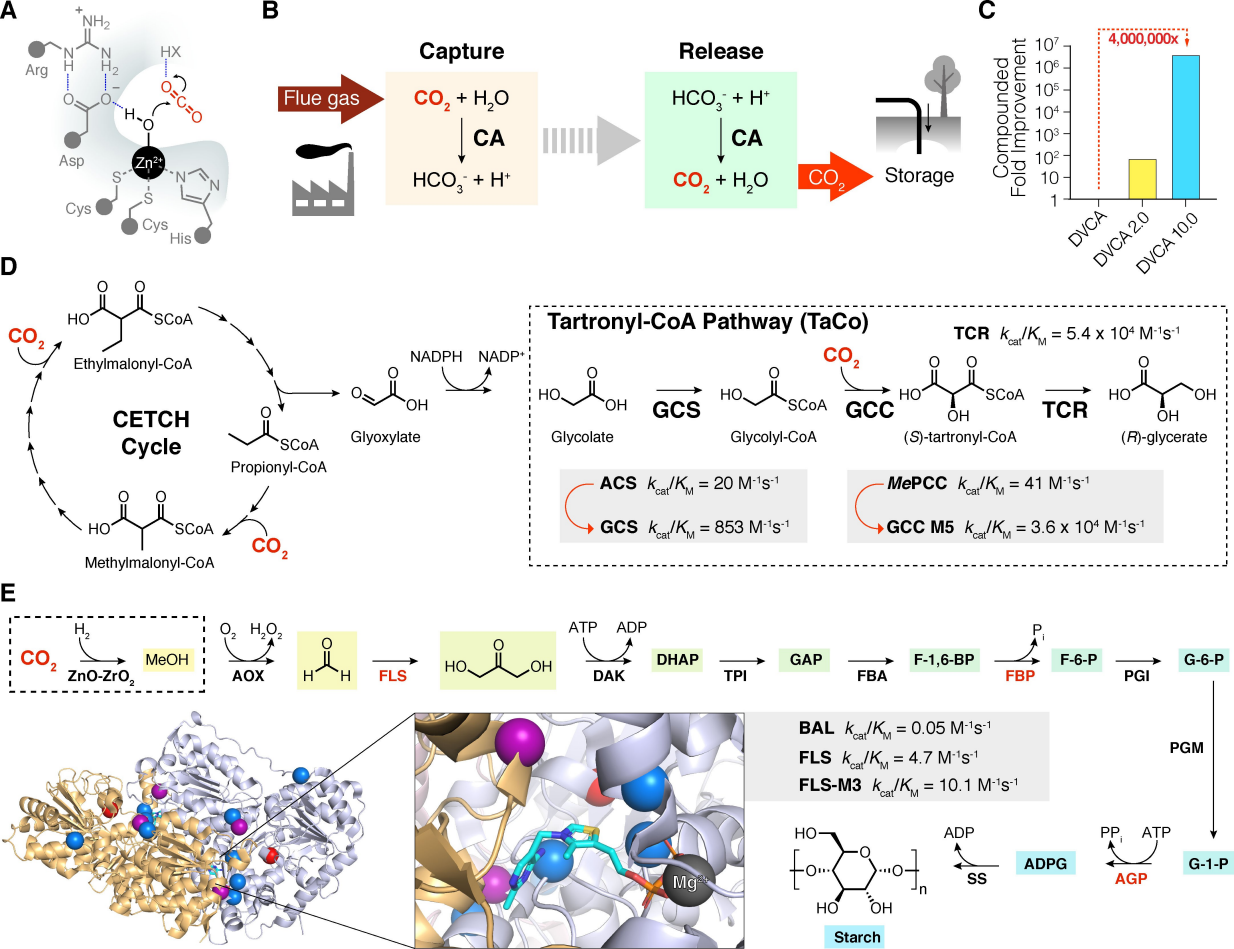
CO_2_ capture and utilisation. A) Schematic active site depiction of a β‐carbonic anhydrase (CA). B) CA can be used as a catalyst in carbon capture and sequestration (CSS) technologies to extract CO_2_ from flue gas as bicarbonate. CA also accelerates CO_2_ release at low temperatures.^[28]^ C) CA from *D. vulgaris* (DVCA) was improved over nine rounds of directed evolution to increase enzyme tolerance to harsh process conditions. Overall, a 4×10^6^‐fold improvement in enzyme performance was achieved.^[28]^ D) The TaCo pathway is a synthetic metabolic pathway for fixation of CO_2_, resulting in the formation of glycerate. This pathway can be interfaced with the CETCH cycle.[^31^, ^32^] (Enzyme and cofactor diagram abbreviations: ACS=acetyl‐CoA synthetase, GCC=glycolyl‐CoA carboxylase, GCS=glycolyl‐CoA synthase, TCR=tartronyl‐CoA reductase, MePCC=*M. extorquens* propionyl‐CoA carboxylase, NADP=nicotinamide adenine dinucleotide phosphate). E) The ASAP pathway is a chemo‐enzymatic cascade reaction for production of starch. Following the chemical reduction of CO_2_ to methanol, a 10‐enzyme cascade converts C1 compounds to starch. Enzymes highlighted in red were identified as bottlenecks in this pathway and subjected to engineering to improve cascade efficiency.^[33]^ The protein structure shows formolase (FLS),^[34]^ which is a computationally designed enzyme (PDB: 4QQ8) based on benzaldehyde lyase (BAL). Blue spheres indicate mutations introduced by computational design, red spheres indicate mutations discovered through mutagenesis and screening, and purple spheres indicate positions that were targeted by computation and mutagenesis. FLS catalysis is mediated by a TPP cofactor, shown in cyan. (Enzyme and cofactor diagram abbreviations: AGP=ADP‐glucose pyrophosphorylase, ADP=adenosine 5′‐diphosphate, AOX=alcohol oxidase, ATP=adenosine 5′‐triphosphate, BAL=benzaldehyde lyase, DAK=dihydroxyacetone kinase, FBA=Fructose‐bisphosphate aldolase, FBP=fructose‐bisphosphatase, FLS=formolase, PGI=phosphoglucose isomerase, PGM=phosphoglucomutase, SS=starch synthase, TPI=triose‐phosphate isomerase, Chemical compound abbreviations: ADPG=ADP glucose, DHAP=dihydroxyacetone phosphate, GAP=D‐glyceraldehyde 3‐phosphate, F‐1,6‐BP=D‐fructose‐1,6‐bisphosphate, F‐6‐P=D‐fructose‐6‐phosphate, G‐6‐P=glucose‐6‐phosphate, G‐1‐P=α‐D‐glucose‐1‐phosphate, P_i_=inorganic phosphate, PP_i_=pyrophosphate).

Carbonic anhydrase (CA) can be used to increase the rate of CO_2_ absorption in aqueous amine solvents and improve the economics of CCS technology. CA is one of Nature's fastest enzymes and promotes the hydration of CO_2_ to bicarbonate and a proton with a turnover number of over 1 million per second (Figure 3A).^[27]^ Unfortunately, wild‐type CAs are poorly tolerant of the harsh alkaline environment of aqueous amine solvents and the elevated temperatures required for CO_2_ desorption. To address this limitation, directed evolution was used to develop an engineered CA that could withstand the alkaline conditions and high temperatures needed for CCS (Figure 3B).^[28]^ A beta‐class CA from *Desulfovibrio vulgaris* (DVCA) was selected as a starting template for engineering due to its high activity in aqueous methyldiethanolamine (MDEA) solvent. A total of 27,000 variants generated through site saturation mutagenesis and recombination of beneficial diversity, were evaluated over nine rounds of directed evolution. The ProSAR algorithm was used to improve screening efficiency during the engineering process. Library members were challenged with incubations at progressively higher temperatures, ranging from 24 h at 42 °C in round one to 1 h at 107 °C in the final stage, prior to activity assessment in the MDEA solvent. The directed evolution campaign afforded a variant, DVCA‐10.0, containing 36 mutations that tolerates temperatures up to 107 °C in the presence of 4.2 M aqueous amine solvent, with a 10,000‐fold improvement in half‐life compared with the parent template (Figure 3C). Furthermore, the engineered DVCA enhanced the rate of CO_2_ absorption 25‐fold compared with a non‐catalysed reaction at pilot plant scale using powerplant‐generated flue gas, where an average of 63.6 % of carbon was captured with no loss in stability recorded over 60 h of operation.

An alternative approach towards a carbon neutral economy is to make use of CO_2_ as a chemical feedstock to produce higher value commodities. Here we can take inspiration from natural carbon fixation pathways that assemble CO_2_ into multi‐carbon organic compounds. However, these pathways are often carbon and energy inefficient. For example, there are few metabolic pathways that allow the direct transformation of C2 compounds such as glycolate and glyoxylate into C3 metabolites, a central process in carbon metabolism. Routes that do exist all result in a loss of carbon via decarboxylation and the release of CO_2_.[^29^, ^30^] Developing new “synthetic” pathways that circumvent carbon and energy loss during glycolate assimilation is therefore of great interest. Towards this goal, inspired by a previously hypothetical tartronyl‐CoA (TaCo) pathway, the Erb lab developed a three‐enzyme carboxylation module for the conversion of glycolate to glycerate (Figure 3D).^[31]^ This pathway fixes CO_2_ instead of releasing it, hence circumventing the carbon loss inherent to other glycolate assimilation pathways.

To make the TaCo pathway a reality, the authors first discovered and rationally engineered a glycolyl‐CoA synthase (GCS) for the conversion of glycolate into glycolyl‐CoA and identified a promiscuous malonyl‐CoA reductase capable of converting tartonyl‐CoA into glycerate (TCR). With these enzymes in hand, attention turned to the development of the key enzyme in the TaCo pathway, a glycolyl‐CoA carboxylase (GCC). After screening a selection of propionyl‐CoA carboxylases for promiscuous activity with glycolyl‐CoA as a substrate, a homolog was identified from *Methylorubrum extorquens* (*Me*PCC). *Me*PCC exhibited low activity for the desired transformation (*k*_cat_=0.01 s^−1^) accompanied by a high ratio of futile ATP hydrolysis compared with tartronyl‐CoA formation (*ca*. 100 : 1). To improve activity, three mutations were introduced into *Me*PCC through rational engineering guided by structural data, leading to a 50‐fold improvement in catalytic efficiency. This triple mutant served as a template for directed evolution using error‐prone PCR coupled with microfluidic screening, monitoring tartronyl‐CoA synthesis via TCR‐mediated reduction of tartronyl‐CoA to glycerate and a concurrent loss in fluorescence due to nicotinamide adenine dinucleotide phosphate (NADPH) reduction. Following introduction of two additional mutations, a GCC M5 variant was identified with a 560‐fold increase in *k*_cat_ compared with the wild‐type enzyme and a 25‐fold reduction in futile ATP hydrolysis. TCR was then combined with the engineered GCS and GCC to assemble the TaCo pathway. Subsequent coupling of the TaCo pathway to the CETCH cycle, a second synthetic CO_2_ fixation pathway,^[32]^ allowed production of the central carbon metabolite glycerate from three molecules of CO_2_. The 17‐enzyme CETCH cycle was first used to produce glyoxylate from CO_2_, which was then converted via a semialdehyde reductase to glycolate that fed into the TaCo pathway, generating 331 μM glycerate at a rate of 4.8 nmol min^−1^ mg^−1^ TaCo enzymes.^[31]^

An alternative chemoenzymatic CO_2_ utilization pathway, the ASAP pathway, was developed by the Ma lab to produce starch, a storage form of carbohydrates and a primary feedstock for bioindustry (Figure 3E).^[33]^ This pathway comprises 11 core reactions, an initial chemical CO_2_ reduction step using an inorganic catalyst to produce methanol, followed by a 10‐enzyme cascade to synthesize starch. For comparison, starch synthesis in plants involves around 60 steps and complex regulation. A key step in this pathway makes use of a computationally designed formolase enzyme (FLS) to catalyse the carboligation of three formaldehyde molecules into dihydroxyacetone (DHA). FLS was derived from the thiamine pyrophosphate (TPP)‐dependent enzyme benzaldehyde lyase (BAL), by installing four mutations into the substrate binding pocket predicted by Rosetta and Foldit calculations followed by introduction of an additional three mutations by directed evolution (Figure 3F).^[34]^

After establishing an initial ASAP 1.0 pathway, three points were identified as bottlenecks. The enzymes involved in the bottlenecks were therefore subjected to engineering to optimize pathway efficiency.^[33]^ Firstly, the catalytic activity of FLS was further improved via additional directed evolution using error prone PCR to produce FLS−M3. The second point of inefficiency was identified as the inhibition of fructose bisphosphatase (FBP), an enzyme mediating the hydrolysis of fructose 1,6‐bisphosphate (F‐1,6‐BP) to fructose 6‐phosphate (F‐6‐P), by the essential cofactors of a later step in the pathway, adenosine 5′‐triphosphate (ATP) and adenosine 5′‐diphosphate (ADP). To resolve this, two sets of previously identified mutations for relieving cofactor and product inhibition were combined to create a four‐point mutant, FBP‐AG^R^, leading to a substantial increase in glucose‐6‐phosphate (G‐6‐P) production. Lastly, the penultimate enzyme in the cascade, ADP‐glucose pyrophosphorylase (AGP) was found to be limited by competition for ATP with an earlier step in the cycle. To increase its competitiveness, three highly active AGPs were constructed by installing previously described amino acid substitutions.[^35^, ^36^] The best variant exhibited a six‐fold increase in starch production from DHA, leading to the improved ASAP 2.0 pathway.^[33]^ Coupling this pathway with a chemical CO_2_ reduction step, using the inorganic ZnO‐ZrO_2_ as a catalyst, and a starch branching enzyme to produce amylopectin as well as amylose (ASAP 3.0), starch could be produced from CO_2_ at a rate of 410 mg liter^−1^ hour^−1^, an 8.5‐fold improvement over natural starch synthesis in maize crops.

## 4 Bioremediation of toxic pollutants

Numerous toxic compounds have been released into the biosphere because of human activities, especially since the industrial revolution. Often these chemicals can bioaccumulate through ecosystems causing a range of negative impacts.^[37]^ However, remarkably, even over relatively short timeframes, microorganisms can begin to adapt to the presence of these anthropogenic contaminants leading to the evolution of enzymes and pathways for their conversion. These enzymes can serve as evolutionary footholds for protein engineers to develop useful bioremediation catalysts to remove toxic compounds and mitigate their detrimental effects.

**Figure 4:**
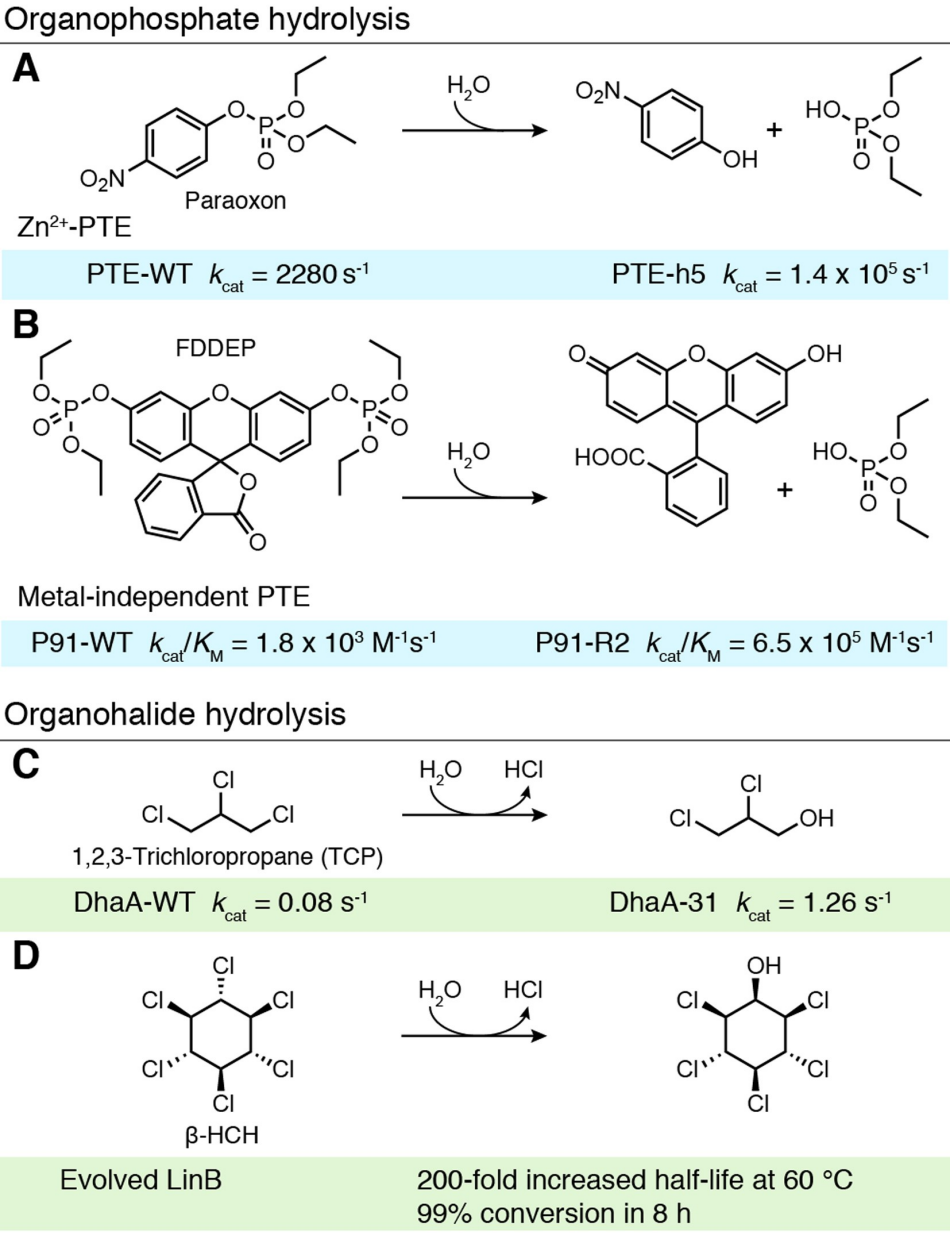
Degradation of pollutants. A) Zn‐dependent phosphotriesterases (PTE) are capable of hydrolysing paraoxon to *p*‐nitrophenol and diethyl‐phosphate. Despite being nearly diffusion controlled, PTE was further improved, giving rise to a 63‐fold increase in *k*_cat_.^[40]^ B) The promiscuous hydrolase P91 shows phosphodiesterase activity towards paraoxon. P91 was further evolved towards a fluorescein‐based model substrate (FDDEP), increasing *k*_cat_/*K*_M_ by 360‐fold.^[42]^ C) *Rhodococcus rhodochrous* haloalkane dehalogenase (DhaA) hydrolyses 1,2,3‐trichloropropane (TCP) to 2,3‐dichloropropane‐1‐ol. Rational engineering in combination with directed evolution yielded DhaA‐31, which displays a 16‐fold increased *k*_cat_ and a 30‐fold increase in enzyme efficiency.^[48]^ D) LinB dehalogenates β‐HCH to pentachlorocyclohexanol. The *T*_m_ of LinB was increased by 23 °C through incorporation of 12 mutations predicted by computation and rational design.^[52]^ (Diagram abbreviations: DhaA=*R. rhodochrous* haloalkane dehalogenase, FDDEP=fluorescein di(diethylphosphate), HCH=hexachlorocyclohexane, WT=wildtype, PTE=phosphotriesterases).

Organophosphates are a common but highly toxic class of pesticides and are a promising target for enzymatic bioremediation.^[38]^ Numerous enzymes have been found to act as phosphotriesterases (PTEs), hydrolysing these pesticides into less toxic products using an active site divalent metal cation.^[39]^ An impressive example of PTE directed evolution was reported by Griffiths and Tawfik, involving a metal‐dependent bacterial PTE that can hydrolyse paraoxon, the active metabolite of the pesticide parathion (Figure 4A).^[40]^ An ultra‐high throughput in vitro compartmentalisation strategy was developed that allows ca. 10^10^ variants to be evaluated from a 50 μ L reaction. Single gene variants were bound to a microbead, translated in a cell‐free manner, and subjected to reaction with a paraoxon derivative, with the produced proteins and reaction products remaining associated with their microbead. Active enzymes were identified by fluorescently labelling microbeads with antibodies that bind the hydrolysed reaction product, followed by sorting of the labelled beads via flow cytometry. Evaluation of a library of 3.4×10^7^ mutated PTE genes allowed selection of a PTE‐h5 variant which displays an impressive *k*_cat_ of 1.4×10^5^ s^−1^ for paraoxon, 63‐times faster than the wild‐type enzyme (PTE‐WT). Interestingly, the promiscuity of bacterial PTEs has also allowed them to be engineered to degrade human nerve agents. Implementation of the computational tool FuncLib, a method which combines Rosetta design calculations and phylogenetic analysis, using PTEs as the input, gave rise to a repertoire of enzymes including one displaying a 122‐fold improved activity towards the toxic nerve agent soman and another showing a 3000‐fold increase in cyclosarin hydrolysis activity.^[41]^

More recently, a metal co‐factor free organophosphate biocatalyst, P91, was discovered by screening millions of metagenomic‐derived sequences for phosphotriesterase activity in picolitre droplets.^[42]^ P91 utilises a Cys‐His‐Asp catalytic triad for catalysis, a motif previously unlinked to phophotriesterase activity. Subsequent evolution of this biocatalyst was carried out in an ultra‐high throughput manner, screening millions of variants by microfluidics.^[43]^ *E. coli* cells expressing libraries of P91 were encapsulated with a lysis agent and a fluorogenic phosphotriester substrate, fluorescein di(diethylphosphate) (FDDEP). Using this approach, the activity of over 600,000 P91 variants could be assessed per hour. Two rounds of evolution produced P91‐R2, a five‐point mutant which exhibited a ≈360‐fold increase in *k*_cat_/*K*_M_ over the wildtype (WT) (P91‐WT *k*_cat_/*K*_M_=1.8×10^3^ M^−1^s^−1^, P91‐R2 *k*_cat_/*K*_M_=6.5×10^5^ M^−1^s^−1^), with an efficiency approaching naturally evolved metal‐assisted phosphotriesterases (Figure 4B).

Organohalide contamination also poses a major environmental challenge, in part due to improper storage and disposal of halogenated organic compounds used in industry and agriculture.^[44]^ One such chemical is 1,2,3‐trichloropropane (TCP), a recalcitrant industrial solvent and suspected human carcinogen (Figure 4C).[^45^, ^46^] DhaA, a haloalkane dehalogenase, has been shown to catalyse cleavage of one of the TCP carbon‐halogen bonds, releasing the less toxic 2,3‐dichloro‐1‐propanol, however its activity levels are modest.^[47]^ A combination of rational design and directed evolution was therefore used to enhance DhaA. In particular, random acceleration molecular dynamics simulations were used to probe the access tunnels connecting the buried active site with the bulk solvent.^[48]^ Focussed libraries were subsequently generated based on these calculations and evaluated for TCP hydrolysis activity. The most active variant identified following screening, DhaA‐31, displayed an impressive 32‐fold increase in activity compared with the parent template (DhaA‐WT *k*_cat_=0.08 s^−1^, DhaA‐31 *k*_cat_=1.2 s^−1^). Interestingly, the introduction of bulky aromatic residues during engineering narrowed the access tunnel to the active site and helped to shield the catalytic centre from bulk solvent. Introducing such evolved enzymes in bacterial metabolism could offer an intriguing route to decontaminating natural environments of TCP. For example, introducing DhaA into the 2,3‐dichloro‐1‐propanol metabolising bacterium *A. radiobacter* AD1, allowed the organism to use TCP as its sole carbon source, converting 3.6 mM TCP completely over 10 days.^[49]^

Beyond their unintentional release into the environment, halogenated compounds such as γ‐hexachlorocyclohexane (HCH, otherwise known as lindane) are also consciously applied to soils and crops as insecticides. Although lindane itself is toxic, its isomeric by‐product, β‐HCH, is perhaps of greater concern as it is particularly recalcitrant and has increased toxicity.^[50]^ The dehalogenase LinB can promote hydrolytic dehalogenation of β‐HCH but has limited applicability due to its poor stability (Figure 4D).^[51]^ To generate a more stable biocatalyst, Floor et al. applied a computational engineering strategy termed FRESCO ‐ framework for rapid enzyme stabilization by computation.^[52]^ FRESCO aims to discover multiple mutations which may have small effects on stability in isolation but result in large beneficial gains when combined. Molecular dynamic simulations are then used to evaluate variants in silico to reduce the number of variants that need to be tested experimentally. The FRESCO‐generated LinB variant contained 12 mutations with a 23 °C higher melting temperature (*T*_m_) than the wildtype and a 200‐fold increased half‐life at 60 °C. The biocatalyst was also substantially more solvent tolerant, with the engineered enzyme able to convert 99 % of β‐HCH dissolved in a DMSO/water mix in 8 h at 45 °C, reaction conditions under which the wildtype enzyme is inactivated within 30 minutes.

Another arm of bioremediation aims to use enzymes to sequester heavy metals often released into the environment during industrial processes. Heavy metals can be carcinogenic, toxic, or radioactive, including metals such as lead, chromium, arsenic, and uranium.^[53]^ One such enzyme is ChrR, a flavoprotein with the nascent ability to reduce chromate Cr (VI) and uranium U(VI). Directed evolution of ChR using error prone PCR afforded a quadruple mutant ChR6, which demonstrated a 300‐fold increase in chromate reduction activity (ChR‐WT *k*_cat_/*K*_M_=4.5×10^4^ M^−1^s^−1^, ChR6 *k*_cat_/*K*_M_=1.3×10^7^ M^−1^s^−1^) along with more modest improvements in uranyl reduction activity.^[54]^ These activity increases were mainly attributed to a single Tyr128Asn mutation. In a subsequent study additional rounds of evolution were performed, making use of the information gained on sequence‐activity relationships to inform a statistical model to predict more active sequences with reduced screening effort.^[55]^ This engineering campaign ultimately delivered a ChR variant, ChrR30, with a 1500‐fold improvement in Cr (VI) reductase activity compared to the wildtype enzyme.

## 5 Deconstruction of plastics

Man‐made plastic materials are an essential commodity in modern day society. However, the negative impacts caused by plastic accumulation in the environment means that new solutions are needed to prevent their release into nature and to circularise the plastic life cycle.[^56^, ^57^, ^58^] Enzymatic depolymerization of plastics has recently emerged as a potentially attractive technology to complement more established mechanical and chemical recycling methods. Biocatalysis offers the potential to selectively hydrolyse plastics into their component monomers, which can then be used to remake new polymers, reducing the need for petroleum‐derived virgin plastics.

**Figure 5:**
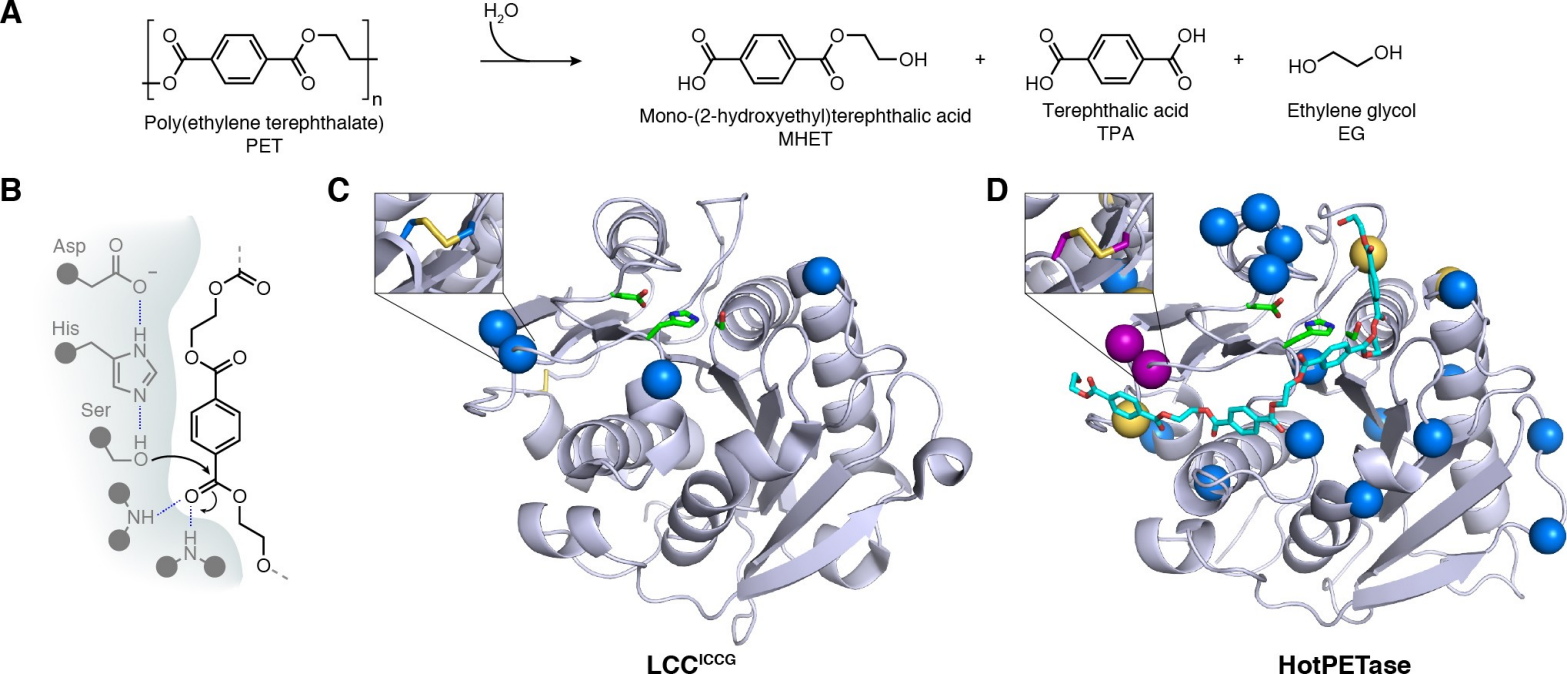
PET depolymerisation. A) Various enzymes capable of hydrolysing PET to MHET, TPA and EG have been discovered to date. B) PET deconstructing enzymes typically belong to the cutinase enzyme family, which are serine hydrolases and utilize a catalytic triad in conjunction with an oxy‐anion hole. C) LCC^ICCG^ is an engineered PETase derived from leaf‐branch compost cutinase.^[69]^ Four mutations (blue spheres) were introduced to increase thermostability (i.e. through installation of a disulphide bridge) (PDB: 7 W44^[75]^). D) HotPETase (model with docked PET substrate (cyan) based on PDB: 7QVH) was engineered for increased thermostability from the natural enzyme *Is*PETase through rational design (yellow & purple spheres) and directed evolution (blue spheres). A total of 24 mutations were introduced leading to a *T*_m_ increase of 34.5 °C.^[75]^ (Diagram abbreviations: PET=poly(ethylene terephthalate), MHET=mono‐(2‐hydroxyethyl) terephthalic acid, TPA=terephthalic acid, EG=ethylene glycol, LCC=Leaf‐branch compost cutinase).

Enzymatic deconstruction of plastics has largely focused on the breakdown of poly(ethylene terephthalate) (PET), an abundant polyester accounting for approximately 10 % of global plastic production.^[57]^ A variety of PET‐hydrolysing enzymes have been reported, including promiscuous cutinases and the naturally evolved *Ideonella sakaiensis* PETase, *Is*PETase.[^59^, ^60^, ^61^, ^62^, ^63^] Despite diverse origins, the general reaction mechanism appears consistent, with enzymes employing a Ser‐His‐Asp catalytic triad to hydrolyse PET ester bonds to release the major soluble products mono‐(2‐hydroxyethyl) terephthalic acid (MHET), terephthalic acid (TPA) and ethylene glycol (EG) (Figure 5A & 5B).^[64]^ However, there are several hurdles that prevent the direct use of such enzymes in commercial processes. Firstly, as PET degrading enzymes have not experienced extensive natural selection pressures for PET deconstruction their intrinsic activities tend to be low. Secondly, the most efficient depolymerisations will occur above the glass transition temperature (*T*_g_) of PET (60–65 °C), where polymer chains become more accessible to enzyme action, which rules out thermally labile proteins. Finally, commercial PET has a significant degree of crystallinity (30–40 %), a form of the polymer which is particularly challenging for enzymes to access.^[65]^

There have been numerous attempts to improve the properties of promiscuous cutinases for PET degradation, with most studies focussing on rational mutagenesis methods. For instance, targeted mutation of a bacterial cutinase from *Thermobifida fusca*, aimed to increase the active site volume to accommodate long polymer chains, while also increasing the affinity of the protein for the hydrophobic PET surface.^[66]^ The engineered variant produced 10‐times more TPA than the starting enzyme. In another example, a cutinase from *Saccharomonospora viridis*, cut190, was rationally mutated, including installation of a disulphide bridge, to increase enzyme stability.^[67]^ The resulting five‐point mutant enzyme could degrade around 30 % of an amorphous PET sample at 70 °C, a three‐fold improvement over WT protein. In a similar vein, point mutations were used to increase the activity of Tfcut2 cutinase by relieving its inhibition by the PET degradation product MHET. The resulting enzyme achieved a 42 % weight loss of amorphous PET film over 50 h, reflecting a 2.7‐fold improvement over the wildtype.^[68]^

One of the most prominent examples of PET biocatalyst engineering is that of Leaf‐branch compost cutinase (LCC), an enzyme discovered from a metagenomics study of compost.^[62]^ LCC is fairly thermostable, with a melting temperature (*T*_m_) of 86.2 °C, however its PET degrading activity required improvement. To improve LCCs activity and thermostability further, protein modelling and molecular docking were employed to identify 11 amino acid positions for mutagenesis.^[69]^ By combining interesting mutations and rationally installing a disulphide bridge, the more thermostable, (*T*_m_ increased by 9.8 °C) and more catalytically active variant LCC^ICCG^ was identified (Figure 5C). The improved characteristics of LCC^ICCG^ enabled its used in a 150 L pilot scale reaction, where 90 % of the PET substrate was depolymerized in around 10 h with a productivity of 16.7 g TPA L^−1^ h^−1^ (200 g TPA produced per Kg of amorphized PET, using 3 mg enzyme per gram of PET), compared to a 53 % conversion achieved with LCC‐WT after 20 h. The resulting TPA was of sufficiently high quality to resynthesise a PET polymer. One potential limitation with the process is the need to amorphise the PET substate prior to enzymatic deconstruction, which increases energy inputs and process costs. Nevertheless, this study has served as the basis for establishing a commercial enzymatic PET recycling process, with a 50,000 ton‐plastic waste‐per‐year biocatalytic recycling plant scheduled to open in 2025.^[70]^

The naturally evolved *Is*PETase has also been subjected to numerous rational engineering attempts.^[71]^ Efforts have generally focused on increasing the enzyme's stability, as wildtype *Is*PETase loses activity after even relatively short periods of incubation at 37 °C.^[72]^ Despite its low thermostability, *Is*PETase is an attractive starting scaffold as it exhibits good PET deconstruction activity under more ambient reaction conditions, unlike the more thermostable cutinases, suggesting that the enzyme potentially has inherently superior catalytic machinery for deconstructing PET.^[63]^ Interestingly *Is*PETase also appears to display greater activity on semi‐crystalline PET materials compared with promiscuous cutinases. One successful effort to rationally mutate *Is*PETase to increase thermostability included the installation of three mutations (S121E, D186H and R280 A), to yield ThermoPETase (*T*_m_=56.8 °C).^[72]^ Other notable attempts to engineer *Is*PETase have involved machine‐learning. For instance, Lu et al., leveraged a structure‐based machine‐learning algorithm, MutCompute,^[73]^ to identify potential stabilizing mutations of *Is*PETase.^[74]^ A variant identified after the addition of two mutations to the ThermoPETase scaffold, yielded FAST‐PETase, which had an increased ability to degrade amorphous PET at temperatures up to 50 °C.

To allow more extensive engineering of *Is*PETase our lab established a high‐throughput and semi‐automated directed evolution pipeline, whereby the PET deconstructing activities of *Is*PETase variants were evaluated by quantifying the release of soluble MHET and TPA products by ultraperformance liquid chromatography (UPLC).^[75]^ The throughput of this engineering platform allowed the screening of over 13,000 variants over the course of evolution, using ThermoPETase as a starting template. Throughout evolution, the selection pressures used were tailored to improve characteristics of interest: initial rounds focused on increasing activity at elevated temperatures by incrementally elevating the PET hydrolysis reaction temperature, next, reaction times were lengthened to enhance enzyme stability during catalytic turnover. Final evolution rounds aimed to identify enzyme variants with an increased propensity for degrading more crystalline (29.8 %) PET samples. Following six rounds of evolution, the resulting 21‐point mutant enzyme, named HotPETase, exhibited a dramatically increased *T*_m_ of 82.5 °C (Figure 5D).^[75]^ HotPETase can efficiently degrade semi‐crystalline PET at temperatures ranging from 40–70 °C, interestingly retaining its low temperature activity unlike the thermostable cutinases. The enzyme is also able to selectively hydrolyse the PET from a PET/PE composite film packaging lid, depolymerising 48.1 % of the PET portion at 60 °C over six days. Intriguingly, evolution also relieved the enzyme‐concentration dependent inhibition seen with wildtype *Is*PETase, despite this not being a direct selection pressure.^[76]^ A current limitation of the enzyme is its operational stability at elevated temperatures. This could potentially be solved by further rounds of engineering with extended reaction times and higher substrate loadings, to mimic commercially relevant conditions.

## 6 Accessing renewable feedstocks and chemicals from biological polymers

Many useful chemicals and feedstocks are currently derived from fossil fuels. To decarbonise our economy, there is a pressing need to find alternative sources of fuels and chemicals, of which generating renewable feedstocks from biomass holds particular promise.[^77^, ^78^] First generation biofuels and bio‐derived chemicals were produced from sugar, starch or oil‐rich crop fermentation.^[79]^ However, using food sources for fuel and chemicals production is likely untenable in the context of a growing population. Focus has therefore shifted to lignocellulosic biomass produced from industrial biomass waste or non‐food energy crops as a potential feedstock.[^80^, ^81^]

Lignocellulosic biomass is a rich source of cellulose, hemicellulose, and lignin; its deconstruction releases a range of products with different useful chemical functionalities. For instance, different polysaccharide products can be produced from degradation of cellulose and hemicellulose, for example D‐glucose which can then be fermented to produce bioethanol or dehydrated into platform chemicals for value added synthesis,[^82^, ^83^] while a range of aromatic compounds are released following deconstruction of lignin. Although many chemical methods exist for the breakdown of lignocellulosic biomass to release these compounds, the abundance of many enzymes, such as cellulases and ligninolytic enzymes, that naturally deconstruct lignocellulose offer a potentially cost‐effective and renewable way to produce biofuels and other chemicals.[^84^, ^85^] However, as lignocellulose is an inherently complex and recalcitrant substrate, meeting these requirements enzymatically is challenging due to slow catalytic rates and low operational stability. Hence, engineering individual lignocellulose deconstructing enzymes has been a topic of intense research. For instance, cellulases have been optimised for improved activity, thermostability and pH tolerance; the details of these efforts are outside the scope of this piece, but many of these efforts are covered in excellent reviews.[^86^, ^87^, ^88^, ^89^] Engineered enzyme cocktails have also been developed, with multiple synergistic biocatalysts working together to deconstruct several biomass components in parallel.^[90]^ These cocktails typically consist of cellulases, cellobiohydrolases, endoglucanases, β‐glucosidases, hemicellulases, and lytic polysaccharide monooxygenases. Prominent examples include the commercial enzyme blend Cellic® CTec3 from Novozyme, a highly efficient mixture of thermostable enzymes for efficiently converting lignocellulosic materials to fermentable sugars with high conversion yields.^[91]^

Although the products of lignocellulose deconstruction and fermentation can be directly used as biofuels, as is the case with bioethanol, there is increasing interest in using the released compounds to produce more diverse products such as biodiesel, surfactants, and lubricants. To this end, Steen et al. aimed to engineer the fatty acid metabolism of *E. coli* to achieve overproduction of fatty acid ethyl esters (FAEEs), subsequently utilising this in a pathway to produce biodiesel from hemicellulose.^[92]^ Metabolic engineering involves the optimisation of enzyme cascades and genetic regulatory processes to promote production of desired substances. In this case, fatty acid biosynthesis from glucose and ethanol was enhanced, eliminating β‐oxidation by deleting *fadE*, whilst promoting FAEE by overexpressing thioesterases, TesAs, and a wax‐ester synthase, AtfA. The need to supply exogenous ethanol was removed by incorporating two *Z. mobilis* genes to allow *E. coli*‐based ethanol production, whilst two further enzymes, an endoxylanase catalytic domain, Xyn10B, from *C. stercorarium* and a *B. ovatus* xylanase, Xsa, fused to *E. coli* OsmY domains were introduced to enable the *E. coli* to grow on hemicellulose, reducing the need to add glucose externally. The resulting strain could hydrolyse biomass derived hemicellulose in the media to xylose, which could then be imported and undergo catabolism using native *E. coli* metabolic pathways. The acetyl‐CoA produced then fed into the engineered fatty acid synthesis pathway to give FAEE‐based biodiesel, with 11.6 mg L^−1^ FAEE formed, demonstrating a consolidated process from biomass to biodiesel in a single organism. Although these yields are currently low, it is likely that further improvements in process efficiency and yields could be achieved by additional engineering of enzymes involved in the pathway.

**Figure 6:**
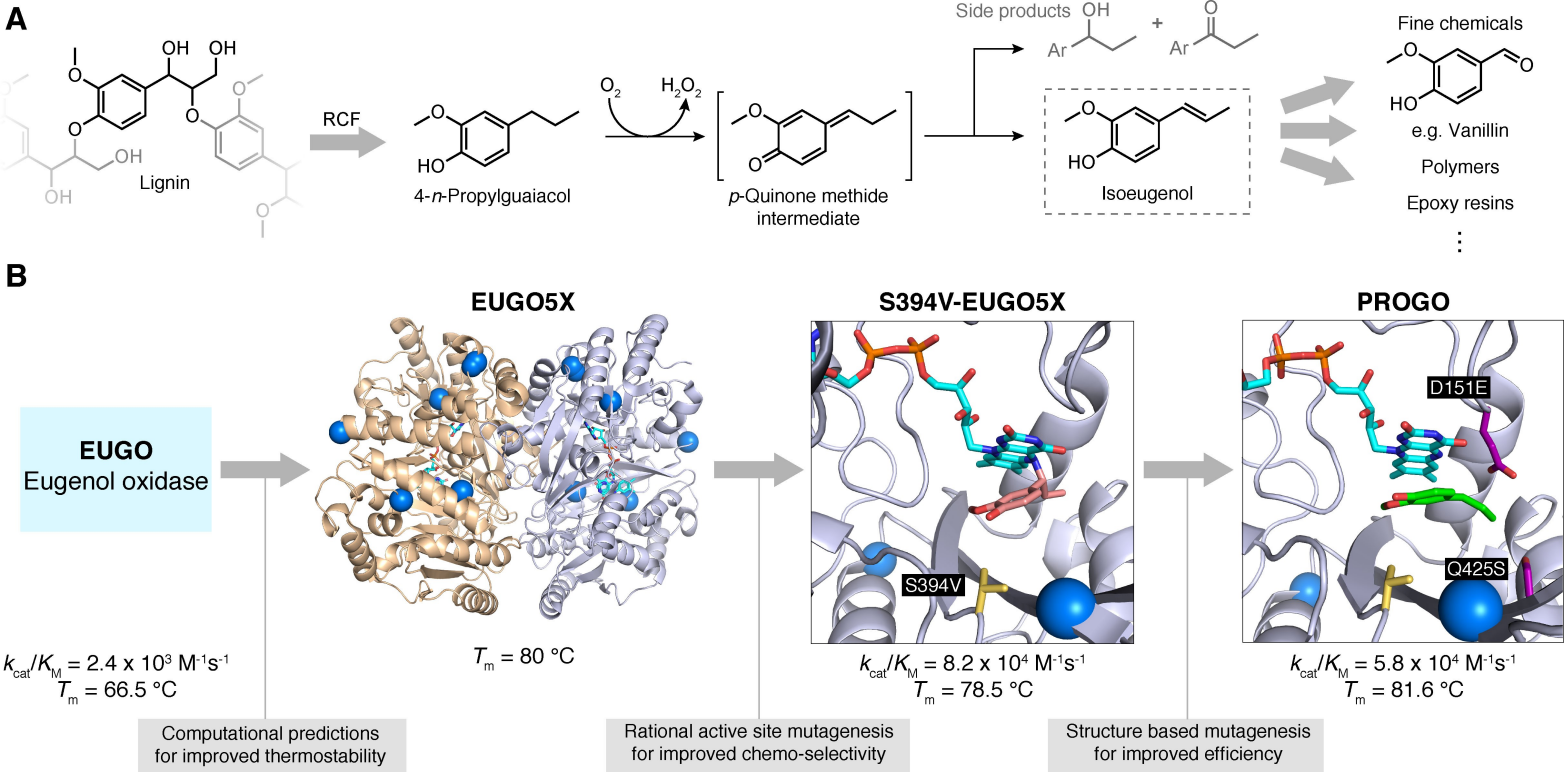
Lignin as a chemical feedstock. A) Lignin can be depolymerised by RCF, yielding 4‐*n*‐propylguaiacol as a main product. VAO‐type oxidases convert 4‐*n*‐propylguaiacol to isoeugenol as well as 4‐(1‐hydroxypropyl)‐2‐methoxyphenol and 1‐(4‐hydroxy‐3‐methoxyphenyl)‐1‐propanone which emerge as side products via hydration of the methide intermediate. Isoeugenol is a versatile precursor for various fine chemicals and polymers. B) Eugenol oxidase (EUGO) was converted into an efficient catalyst for isoeugenol production, by introducing mutations that improve thermostability (blue spheres), chemo‐selectivity (S394 V‐EUGO5X: yellow sticks) and to protect the FAD cofactor (cyan sticks) by preventing adduct formation with the substrate (PROGO ‐ D151E & Q425S: purple sticks).^[95]^ The crystal structure of S394 V‐EUGO5X (PDB: 7YWU) shows the formation of a covalent adduct between the substrate (salmon) and FAD, which is absent in the PROGO crystal structure (PDB: 7YWV, substrate shown in green). (Diagram abbreviations: FAD= flavin adenine dinucleotide, RCF=reductive catalytic fractionation).

As indicated above, lignin is a potentially rich source of valuable phenolic compounds, and new emerging technologies such as reductive catalytic fractionation (RCF) are being developed to release these useful monomers from the polymer.[^78^, ^93^] However, the major products of this process, like 4‐*n*‐propylguaiacol, remain largely unexploited as chemical starting materials.^[94]^ To address this, Guo et al. aimed to design a biochemical route to produce isoeugenol from 4‐*n*‐propylguaiacol,^[95]^ a useful starting point for the synthesis of numerous commodity chemicals including vanillin and epoxy resins (Figure 6A).[^96^, ^97^] Eugenol oxidase (EUGO) was selected as the template for engineering, but initially displayed low activity for the desired dehydrogenation of 4‐*n*‐propylguaiacol along with competing side reactions including benzylic oxidation. Initial engineering efforts focussed on increasing enzyme stability, using the previously mentioned FRESCO algorithm (Figure 6B). The resulting mutant EUGO5X had a 15 °C increase in *T*_m_. With a thermostable scaffold in hand, attention turned to improving chemoselectivity for isoeugenol production. Using computational protein‐ligand docking, a small library of 16 single point mutations was predicted. The best of these, S394 V‐EUGO5X, produced the highest proportion of isoeugenol (80 %) versus other undesired reaction by‐products. Although a significant improvement over the wildtype, S394 V‐EUGO5X still exhibited a low *k*_cat_ of 0.028 s^−1^, which could be attributed to the generation of a slowly decaying covalent flavin adenine dinucleotide (FAD) adduct in the enzyme active site. Hence, residues close to where the FAD bound were targeted for mutation to destabilise the adduct. This strategy proved to be very successful, affording the final variant PROGO (4‐*n*‐propylguaiacol oxidase) that could convert ≈80 % of the starting material to isoeugenol in 3 h whilst retaining the beneficial characteristics installed during previous engineering rounds. In a preparative scale reaction with 1.27 g 4‐*n*‐propylguaiacol substrate, isoeugenol was produced in 42 % yield using *E. coli* whole cells expressing PROGO.

## 7 Summary and Outlook

Designing, replacing, or supplementing processes with biocatalytic strategies powered by engineered enzymes offers a promising avenue to a more sustainable future. The examples featured in this review showcase how directed evolution can produce optimized biocatalysts with the potential to radically change how we conduct many practices for the better. In some cases, engineered enzymes have already reached, or appear close to, commercial utility, such as those for carbon fixation, plastic recycling, and lignocellulose deconstruction. However, despite these successes, there are still some limitations which must be overcome for biocatalysis and enzyme engineering to be adopted more widely for sustainability applications. For instance, there are numerous processes for which no enzymes are available, or where protein engineering is difficult. Key examples include the development and implementation of biocatalytic recycling strategies for alternative plastics such as nylons, and bioremediation strategies to clear other toxic and environmentally recalcitrant chemicals such as polyfluoroalkyl substances (PFAS). Additionally, many of the examples described have yet to be executed at scale under industrially relevant conditions, and it is unclear how biocatalytic processes would be used in the context of existing infrastructure. Finally, in some cases enzymatic approaches may not be commercially competitive or offer the intended sustainability advantages.

To address these challenges, new methods for enzyme discovery, design, and optimization, including those based on deep learning, hold great promise for quickly expanding the range of chemistries accessible.[^41^, ^98^, ^99^] Advanced ultra‐high throughput screening assays will also speed up enzyme discovery and engineering campaigns,^[100]^ while cutting‐edge analysis methods can enable the rapid identification of challenging analytes.^[101]^ To scale up reactions and improve feasibility, pilot or industrial scale process tests need to be conducted, which should be coupled with additional rounds of protein optimization to correct any short falls.^[102]^ Exploring the integration of biocatalysis with new or existing complementary chemical approaches can further improve process efficiency and sustainability, allowing us to take advantage of the most beneficial aspects of different technologies.[^103^, ^104^] Finally, it is important to verify the sustainability gains and cost implications of supplanting current processes with biocatalytic alternatives using life cycle and techno‐economic analyses.[^105^, ^106^] The outputs of these assessments are also crucial to define commercially viable and environmentally sustainable process operating conditions to guide target parameters for enzyme engineering. Humanity requires new approaches and ideas to swiftly address the environmental problems facing our planet. We are optimistic that biocatalysis can play an important role in addressing these challenges, helping us reach our sustainability goals while protecting our environment.

## Conflict of interest

The authors declare no conflict of interest.

8

## Biographical Information

*Following his PhD in synthetic organic chemistry under the supervision of E. J. Thomas, Anthony carried out postdoctoral research with N. J. Turner & S. L. Flitsch based in the Manchester Institute of Biotechnology, and subsequently with D. Hilvert at ETH Zurich. Anthony started his independent research career in 2016 based in the Manchester Institute of Biotechnology at the University of Manchester, where he is a professor of organic and biological chemistry. His research interests lie in the design and evolution of enzymes with new function*.

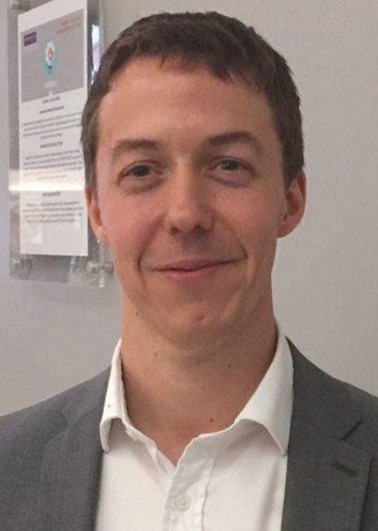

## Biographical Information

*Elizabeth L. Bell is a Postdoctoral researcher at the National Renewable Energy Laboratory (NREL) in Colorado, USA. After completing a BA in Natural Sciences and MPhil at St Catharine's College, University of Cambridge, UK, Elizabeth obtained her Ph.D. in the group of Prof. Anthony P. Green, at the University of Manchester, UK, conducting research into the directed evolution of PET plastic degrading enzymes. Following on from a post‐doc with A. P. Green engineering carbon fixing enzymes, Elizabeth's current research at NREL focusses on discovering and engineering enzymes for the deconstruction of nylons and polyurethanes*.

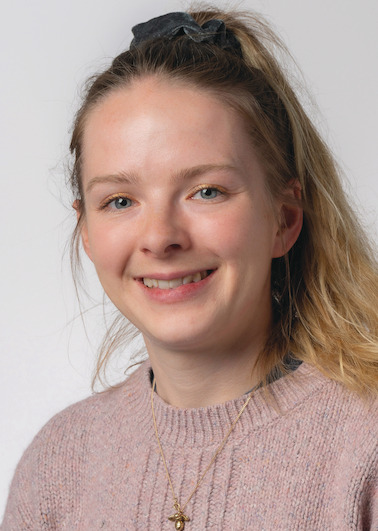

## Data Availability

Data sharing is not applicable to this article as no new data were created or analyzed in this study.
